# Static Magnetic Fields Enhance the Chondrogenesis of Mandibular Bone Marrow Mesenchymal Stem Cells in Coculture Systems

**DOI:** 10.1155/2021/9962861

**Published:** 2021-11-27

**Authors:** Ming Zhang, Weihao Li, Wei He, Yanhua Xu

**Affiliations:** ^1^Department of Orthodontics, School of Stomatology, Kunming Medical University, Kunming, Yunnan 650500, China; ^2^Yunnan Key Laboratory of Stomatology, Kunming Medical University, Kunming, Yunnan 650500, China

## Abstract

**Objectives:**

Combining the advantages of static magnetic fields (SMF) and coculture systems, we investigated the effect of moderate-intensity SMF on the chondrogenesis and proliferation of mandibular bone marrow mesenchymal stem cells (MBMSCs) in the MBMSC/mandibular condylar chondrocyte (MCC) coculture system. The main aim of the present study was to provide an experimental basis for obtaining better cartilage tissue engineering seed cells for the effective repair of condylar cartilage defects in clinical practice.

**Methods:**

MBMSCs and MCCs were isolated from SD (Sprague Dawley) rats. Flow cytometry, three-lineage differentiation, colony-forming assays, immunocytochemistry, and toluidine blue staining were used for the identification of MBMSCs and MCCs. MBMSCs and MCCs were seeded into the lower and upper Transwell chambers, respectively, at a ratio of 1 : 2, and exposed to a 280 mT SMF. MBMSCs were harvested after 3, 7, or 14 days for analysis. CCK-8 was used to detect cell proliferation, Alcian blue staining was utilized to evaluate glycosaminoglycan (GAG), and western blotting and real-time quantitative polymerase chain reaction (RT-qPCR) detected protein and gene expression levels of SOX9, Col2A1 (Collagen Type II Alpha 1), and Aggrecan (ACAN).

**Results:**

The proliferation of MBMSCs was significantly enhanced in the experimental group with MBMSCs cocultured with MCCs under SMF stimulation relative to controls (*P* < 0.05). GAG content was increased, and SOX9, Col2A1, and ACAN were also increased at the mRNA and protein levels (*P* < 0.05).

**Conclusions:**

Moderate-intensity SMF improved the chondrogenesis and proliferation of MBMSCs in the coculture system, and it might be a promising approach to repair condylar cartilage defects in the clinical setting.

## 1. Introduction

Clinically observed cartilage damage of the temporomandibular joint mainly refers to the damage to the functional surfaces of condyles and joints. If not treated in a timely fashion, it is very likely to induce temporomandibular joint osteoarthritis (TMJOA). Patients with TMJOA often suffer from pain in the joint area, limiting mouth opening and precipitating mandibular movement disorder [1], which seriously reduces their quality of life. As articular cartilage has no blood vessels, no lymph, no nerves, and low metabolic activity, once damage occurs, it is difficult to repair [2]. Currently, the treatment of condylar cartilage defects is mainly conservative, such as physical therapy, occlusal guide plate, nonsteroidal anti-inflammatory drugs, and joint puncture [3]. Surgical interventions, such as allograft and chondrocyte transplantation, are appropriate for patients with severe symptoms [4]. Although the above treatments can block the progression of the disease to a certain extent, problems such as immune rejection and high medical costs remain [5]. However, there is still no effective reconstruction method suitable for repairing the defective area.

The development of cartilage tissue engineering offers a possibility for the repair of condylar cartilage defects. Seed cells form the basis of tissue engineering research [6]. Theoretically, autologous chondrocytes are ideal seed cells, but their clinical application is limited by the difficulty of harvesting them, their limited proliferative capacity, and their tendency to dedifferentiate [7]. In contrast, bone marrow mesenchymal stem cells (BMSCs) have become the most promising seed cell source due to their multidirectional differentiation potential and low immunogenicity [8]. The induction of BMSC chondrogenesis to repair articular cartilage defects has recently begun to be intensively studied [9]. Many studies focused on BMSCs from the femur and tibia [10]; however, few studies were interested in the mandibular BMSCs, which contribute to mandible development. At the 7^th^ week of the embryo [11], MBMSCs located at the base of the mandible gradually coagulated into a mass, and then the central cells of the mass differentiated into osteoblasts, finally forming the mandible by intramembrane osteogenesis. Besides, MBMSCs need shorter-term primary culture and differentiation time [12] and exhibit stronger proliferation and antiapoptotic potential as compared to long-bone BMSCs [13], which suggests that they may be a better seed cell source for condylar cartilage regeneration.

In recent years, coculture techniques have been proven to be powerful tools in cartilage tissue engineering to elucidate cellular interactions so as to guide and support chondrogenesis [14]. Compared with single-cell cultures, the coculture system has shown its unique advantages with the biomimetic in vivo microenvironment [15]. The Transwell chamber facilitates the observation of cell growth and the investigation of cell-cell interactions via soluble mediators. A ratio of 2 : 1 of BMSCs/chondrocytes in the Transwell indirect coculture system was optimal to induce BMSC chondrogenesis by chondrocytes [16, 17]. In addition, chondrocytes included in the coculture system not only induce the chondrogenic differentiation of BMSCs [18] but also reduce the calcification and hypertrophy phenotypes that arise during differentiation [19]. In terms of mechanical properties and biochemical parameters, engineered cartilage produced from BMSC/chondrocyte cocultures was proven to be superior to that produced from BMSCs by growth factors [20].

Meanwhile, stem cell chondrogenesis is also influenced by physical stimulation, as well as the magnetic force, which has excellent prospects for application as a noninvasive physical stimulation [21]. Substantial evidence indicated that the strength of magnetic fields should preferably be kept in the medium range (1 mT to 1 T) [22], and moderate-intensity SMF are capable of biologically influencing cells. SMF consist of permanent magnets that are stable in direction and intensity [23], and it can fluctuate the intracellular magnetic flux, then affect transmembrane protein signal transduction, and induce signal cascade reactions, ultimately changing the structure and function of cells [24]. Furthermore, SMF also had been proved to play a significant role in chondrogenesis. Moderate-intensity SMF can effectively promote the proliferation of BMSCs and enhance their chondrogenic differentiation [25] through a transforming growth factor-*β*- (TGF-*β*-) dependent signaling pathway [26]. However, the role of SMF on chondrogenesis of BMSCs is still unknown in coculture systems. Therefore, the aim of this research is to investigate the effect of a moderate-intensity SMF on the chondrogenesis and proliferation of MBMSCs in the MBMSC/MCC Transwell coculture system. It will provide a scientific basis for obtaining better cartilage tissue engineering seed cells and a hope for the effective repair of condylar cartilage defects in clinical practice.

## 2. Materials and Methods

### 2.1. Isolation, Culture, and Identification of MCCs

**Figure 1:**
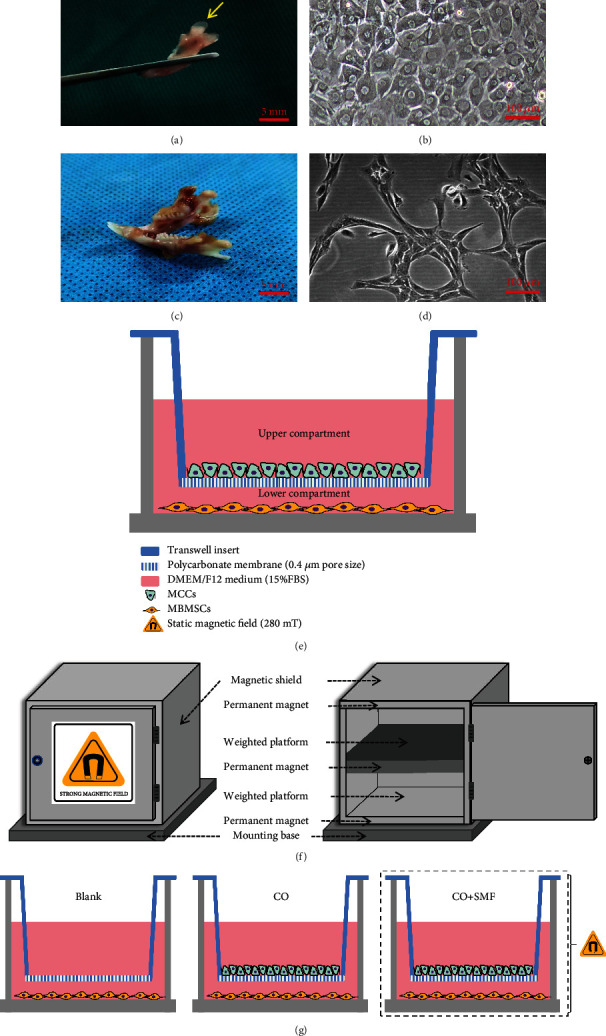
Transwell indirect coculture system and 280 mT moderate-intensity SMF loading device. (a) Isolated condylar cartilage. (b) MCCs cultured to the 2nd passage. Amplification = 10x, scale bar = 100 *μ*m. (c) Isolated mandibles. (d) The 3rd passage of the purified MBMSCs. Amplification = 10x, scale bar = 100 *μ*m. (e) 280 mT SMF device. (f) Establishment of the Tanswell indirect coculture system. (g) Experimental grouping.

Supported by the Model Animal Research Center of Kunming Medical University, condylar cartilage tissues (Figure 1(a)) of 2-day-old SD (Sprague Dawley) rats (male, 5 g) were cut and digested with 0.25% trypsin (Gibco Invitrogen, USA) at 37°C for 20 min, then with 0.2% collagenase type II (Sigma Aldrich, USA) at 37°C for 1 h [27]. The cells were incubated in the DMEM/high glucose medium (BI, Israel) containing 10% fetal bovine serum (Gibco Invitrogen) (Figure 1(b)). MCCs (2 × 10^4^ cells/ml) were fixed with 4% paraformaldehyde when they reached 70% confluence. Immunocytochemistry was performed as follows: cells at the 1^st^ passage were incubated with 0.4% Triton X-100 for 30 min, blocked with 3% H_2_O_2_ for 15 min, and incubated with 10% goat serum for 30 min, and primary antibodies (anti-Col2A1, 1 : 50 dilution; Abcam, USA) were added at 4°C overnight. Secondary antibodies (Goat Anti-Rabbit IgG, 1 : 200 dilution; Affinity Biosciences, USA) were added for 1 h, stained with the DAB substrate kit (MXB, Fuzhou, China), and restained with hematoxylin, and the slides were sealed after gradient ethanol dehydration and air-drying. Toluidine blue staining (Solarbio, Beijing, China) was performed for 30 min, rinsing with running water and air-drying.

### 2.2. Isolation, Culture, and Identification of MBMSCs

Derived from 3-week-old SD rats (male,70 g, purchased from the Model Animal Research Center of Kunming Medical University), the mandibles (Figure 1(c)) were removed and the medullary cavity was exposed [28]. The medullary cavity was rinsed out using a 1 ml syringe loaded with the SD rat BMSC culture medium (Cyagen Biosciences, USA), and the rinse solution was transferred to a culture flask [29, 30]. The cells were incubated in the DMEM/F-12 medium (HyClone, USA) containing 15% fetal bovine serum (Gibco Invitrogen) and purified by limiting dilution analysis (Figure 1(d)).

The 3^rd^-passage MBMSCs were collected, washed with phosphate-buffered saline (PBS) (supplemented with 2% FBS) to remove cell debris, resuspended in 1 ml of PBS (supplemented with 2% FBS), and adjusted to 1 × 10^7^ cells/ml. One hundred *μ*l of cell suspension was pipetted into a centrifuge tube, and antibodies (CD29, CD90, CD44, CD11b, CD34, and CD45; BD Biosciences, USA) were added. Tubes were incubated in the dark for 30 min at 4°C. Samples were analyzed on an LSR II Flow Cytometer (BD Biosciences, USA). Detection and analysis was conducted after setting appropriate gates. Osteogenic, lipogenic, or chondrogenic differentiation of MBMSCs was induced using the SD Rat MBMSC Differentiation Medium (Cyagen Biosciences) according to the instructions, and then they were stained with alizarin red, oil red O, or alizarin blue. MBMSCs (1 × 10^3^) were seeded into 60 mm culture dishes, and crystal violet staining was performed when the cells showed obvious colonization. The clone formation rate was calculated as follows: number of clones/number of cells seeded × 100%.

### 2.3. Establishment of the Transwell Indirect Coculture System

The 3^rd^-passage MBMSCs and 2^nd^-passage MCCs were harvested. The MBMSCs were seeded into the lower chambers of Transwell plates (0.4 *μ*m pore size; Corning Life Sciences, USA) and the MCCs into the upper chambers at a ratio of MBMSCs : MCCs of 1 : 2. The cells were cultured in the DMEM/F-12 medium (HyClone) supplemented with 15% FBS (Gibco Invitrogen). The free movement of culture media in the upper and lower chambers established the Transwell indirect coculture system between MBMSCs and MCCs (Figure 1(e)).

### 2.4. Generation of a 280 mT SMF

For in vitro stimulation of the cells by exposure to a 280 mT SMF, a NdFeB generator (Figure 1(f)) was placed in the incubator. The structure of it includes a mounting base, 280 mT NdFeB permanent magnet, weighted platform, and magnetic shield. Up to 12 culture plates can be placed on the weighted platform. Between the two weighted platforms are NdFeB permanent magnets, which generate SMF in the vertical direction and then act on the culture plate. The magnetic shield is made of galvanized iron sheet. The outer layer of the mounting base is plated with antimagnetic stainless steel to isolate the magnetic field. A constant magnetic field of 280 mT was confirmed using an HT20 Digital Teslameter (Hengtong, Suzhou, China). Experimental groups (Figure 1(g)) were as follows: (1) blank group (Blank) with single MBMSCs, (2) control group (CO) with MBMSCs cocultured with MCCs, and (3) experimental group (CO+SMF) with MBMSCs cocultured with MCCs under SMF stimulation.

### 2.5. Cell Proliferation Assay

The 3^rd^-passage MBMSCs and 2^nd^-passage MCCs were harvested, and the lower chambers of the Transwell 24-well plates were inoculated with 3 × 10^3^ MBMSCs, while the upper chambers of the CO and CO+SMF groups were inoculated with 6 × 10^3^ MCCs, and the CO+SMF group was incubated with exposure to the 280 mT SMF. Four replicates were included for each group. According to the manufacturer's instructions for the Cell Counting Kit-8 (CCK-8, Dojindo Molecular Technologies, Kumamoto, Japan), on days 1, 3, 5, 7, 9, and 11, 300 *μ*l of the prepared CCK-8 solution was added to each well and incubated at 37°C for 1.5 h in the dark. 100 *μ*l of the medium was taken from each well and transferred to a 96-well plate, and the optical density (OD) of each well at 450 nm was measured and recorded using a SpectraMax M5 microplate reader (Molecular Devices, CA, USA).

### 2.6. Alcian Blue Staining

The 3^rd^-passage MBMSCs and 2^nd^-passage MCCs were harvested, and the lower chambers of the Transwell 24-well plate were inoculated with 2 × 10^5^ MBMSCs, while the upper chambers of the CO and CO+SMF groups were inoculated with 4 × 10^5^ MCCs, and the CO+SMF group was incubated in the 280 mT SMF. The GAG content of MBMSCs from the three groups was identified by Alcian blue staining on days 3, 7, and 14 using a standard Alcian blue staining kit (pH = 2.5) (Solarbio). Cells were washed 3x with PBS, fixed with 4% paraformaldehyde at room temperature for 20 min, again washed 3x with PBS (3 min each time), incubated with hydrochloric acid solution for 3 min, stained with Alcian staining solution for 30 min, and rinsed with running water. The images were acquired using an inverted microscope with consistent photographic parameters.

### 2.7. Western Blot Analysis

Total proteins were extracted from each group of MBMSCs on days 3, 7, and 14 using the RIPA tissue/cell lysis buffer (Solarbio). Protein concentration was determined with a BCA kit (Beyotime, Shanghai, China). The proteins were separated by sodium dodecyl sulfate-polyacrylamide gel electrophoresis at 80 V for 30 min followed at 120 V for 1 h, then transferred to polyvinylidene difluoride membranes (Millipore, USA), and blocked with Western blocking solution (Beyotime). The membrane was incubated with primary antibodies (anti-SOX9, 1 : 1000 dilution; anti-Col2A1, 1 : 1000 dilution; anti-Aggrecan, 1 : 1000 dilution; and anti-*β*-actin, 1 : 10,000 dilution; Abcam, USA) at 4°C overnight, followed by incubation with secondary antibodies (HRP Goat Anti-Rabbit IgG, 1 : 2000 dilution; Abcam) for 1 h. The ECL solution (Beyotime) was used for visualization and the Bio-Rad ChemiDoc™ XRS system for imaging, and the signal was captured and analyzed using Image Lab™ software (Bio-Rad).

### 2.8. RT-qPCR

Total RNA from each group of MBMSCs was isolated on days 3, 7, and 14 using RNA Extraction Kits (Takara, Kyoto, Japan). cDNA was then obtained by reverse transcription using the PrimeScript RT Reagent Kit with gDNA Eraser (Takara) according to the manufacturer's instructions. qPCR was performed in a Real-Time PCR System (Thermo Fisher Scientific, USA) using TB Green® Premix Ex Taq™ II (Takara), according to the manufacturer's instructions. The relative mRNA level (fold change) was then calculated, and expressions were normalized to the levels of the internal reference gene *β*-actin. The primers (*SOX9*, *Col2A1*, *ACAN*, and *β*-*actin*) were designed and synthesized by Sangon Biotech (Shanghai, China). The sequences were as follows: *SOX9* forward, 5′-TGGCAGAGGGTGGCAGACAG-3′ and reverse, 5′-CGTTGGGCGGCAGGTATTGG-3′; *Col2A1* forward, 5′-GGAGCAGCAAGAGCAAGGAGAAG-3′ and reverse, 5′-GGAGCCCTCAGTGGACAGTAGAC-3′; *ACAN* forward, 5′-GCTACGACGCCATCTGCTACAC-3′ and reverse, 5′-ATGTCCTCTTCACCACCCACTCC-3′; and *β*-*actin* forward, 5′-ACAGCTTCACCACCACAGCT-3′ and reverse, 5′-GAGGAAGAGGATGCGGCAGT-3′.

### 2.9. Statistical Analysis

The results are expressed as means ± standard deviation (SD) and analyzed by SPSS 19.0 software. Each experiment was repeated 3 times. Data from different groups were compared using one-way ANOVA. The difference was considered statistically significant when the *P* value was performed <0.05.

## 3. Results

### 3.1. Identification of MCCs

**Figure 2:**
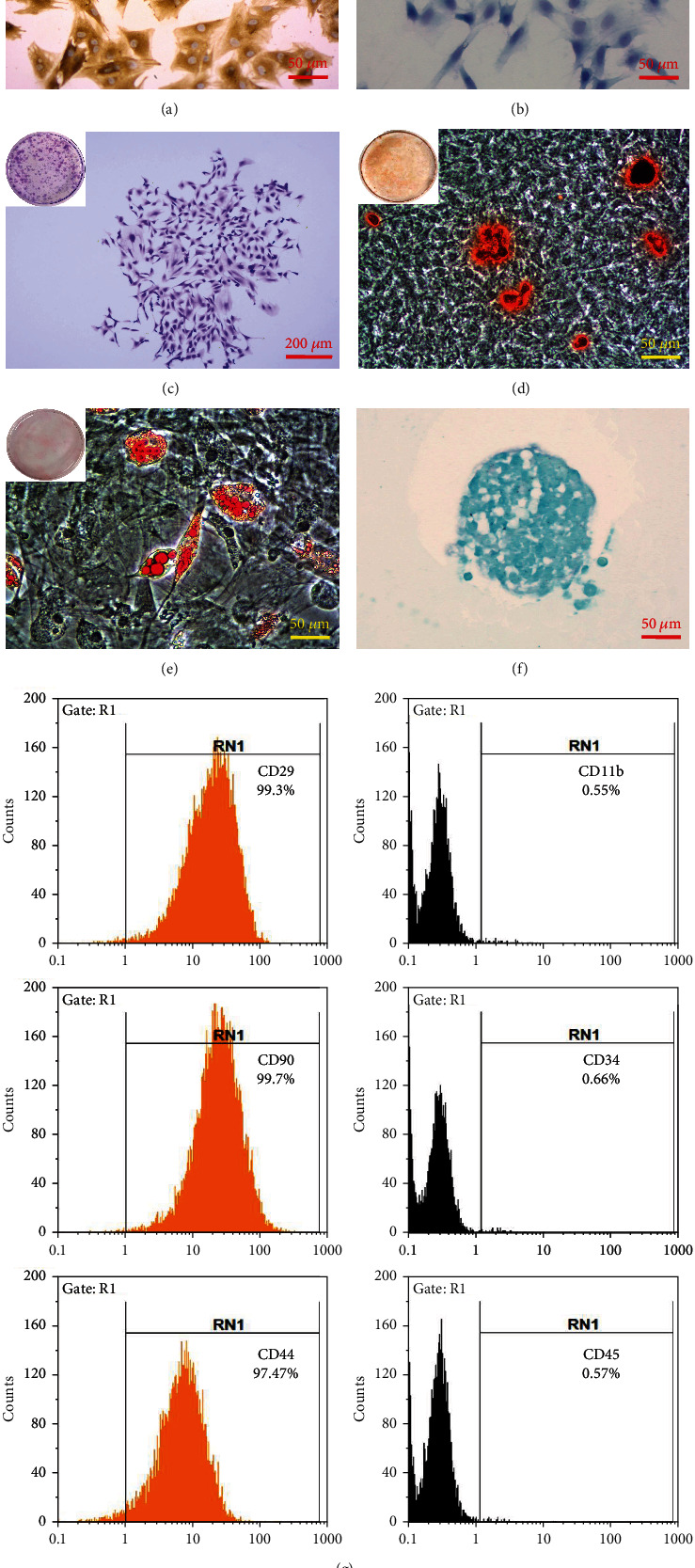
Identification of MCCs and MBMSCs. (a) Immunocytochemical staining of MCCs for Col2A1. Amplification = 20x, scale bar = 50 *μ*m. (b) Toluidine blue staining of MCCs. Amplification = 20x, scale bar = 50 *μ*m. (c) Crystal violet staining of MBMSCs. Cloning efficiency was 35%. Amplification = 4x, scale bar = 200 *μ*m. (d) Alizarin red staining of osteogenic-induced MBMSCs. Amplification = 20x, scale bar = 50 *μ*m. (e) Oil red O staining of lipogenic-induced MBMSCs. Amplification = 20x, scale bar = 50 *μ*m. (f) Alcian blue staining of MBMSCs after cartilage induction. Amplification = 20x, scale bar = 50 *μ*m. (g) Flow cytometry identification of typical surface markers of MBMSCs.

Immunocytochemistry and toluidine blue staining were performed to identify the MCCs we had obtained. These cells were strongly positive for Col2A1, with a brownish-yellow color in the cytosol and cytoplasm and light blue nuclei (Figure 2(a)), indicating that they were capable of secreting Col2A1. Toluidine blue staining revealed blue-purple coloration of the cytoplasm and dark blue nuclei (Figure 2(b)), indicating that the cells were able to synthesize and secrete proteoglycans. The above results document that MCCs obtained by the combined enzymatic digestion method used here can synthesize and secrete large amounts of Col2A1 and proteoglycans and that they are biologically intact.

### 3.2. Identification of MBMSCs

To identify the obtained MBMSCs, we tested their multidirectional differentiation ability and proliferative capacity. The cloning efficiency of MBMSCs was calculated to be 35% by crystal violet staining and colony formation enumeration by counting under the microscope (Figure 2(c)). After 21 days of osteogenic induction, red calcified nodules were observed by alizarin red staining (Figure 2(d)); after 28 days of lipogenic induction, a large number of lipid droplets were observed in the cytoplasm (Figure 2(e)); and after 28 days of chondrogenic induction, a large amount of acid mucopolysaccharide was observed by Alcian blue staining (Figure 2(f)). The flow cytometry results showed that the isolated MBMSCs expressed typical surface markers of MBMSCs (CD29, CD90, and CD44) but not CD11b, CD34, or CD45 (Figure 2(g)). The above results indicate that the MBMSCs we obtained are robust and pure and have multidirectional differentiation potential, consistent with the functional characteristics of MBMSCs.

### 3.3. Effects of an SMF on the Proliferation of MBMSCs in a Coculture System

**Figure 3:**
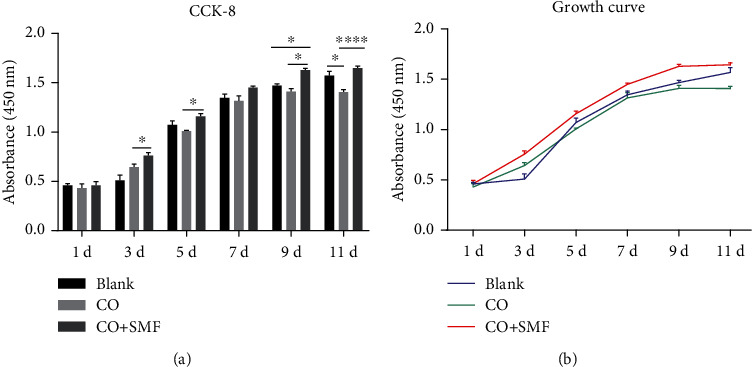
Effect of an SMF on the proliferative capacity of MBMSCs in the coculture system. (a) The proliferative activity of MBMSCs in each group. (b) The growth curves of MBMSCs in each group. ^∗^*P* < 0.05, ^∗∗∗∗^*P* < 0.0001. Each group repeated three times (*n* = 3). Data presented as means ± SD.

The results of the CCK-8 proliferation test showed that the OD values of each group increased gradually with time, reaching a stationary phase on day 11 (Figures 3(a) and 3(b)). Compared with the blank group, the OD value of the control group was suppressed on days 1, 5, 7, and 9 (*P* > 0.05) and significantly decreased on day 11 (*P* < 0.05), and the OD values of the experimental group showed an increasing trend from day 3 to day 11, which was statistically significant on day 9 (*P* < 0.05). Compared with the control group, the OD values of the experimental group showed an increasing trend on days 3, 5, 9 (*P* < 0.05), and 11 (*P* < 0.0001). The above results of the proliferation assay showed that 280 mT moderate-intensity SMF promoted the proliferation of MBMSCs in the coculture system.

### 3.4. Effect of an SMF on MBMSC Chondrogenesis in the Coculture System

**Figure 4:**
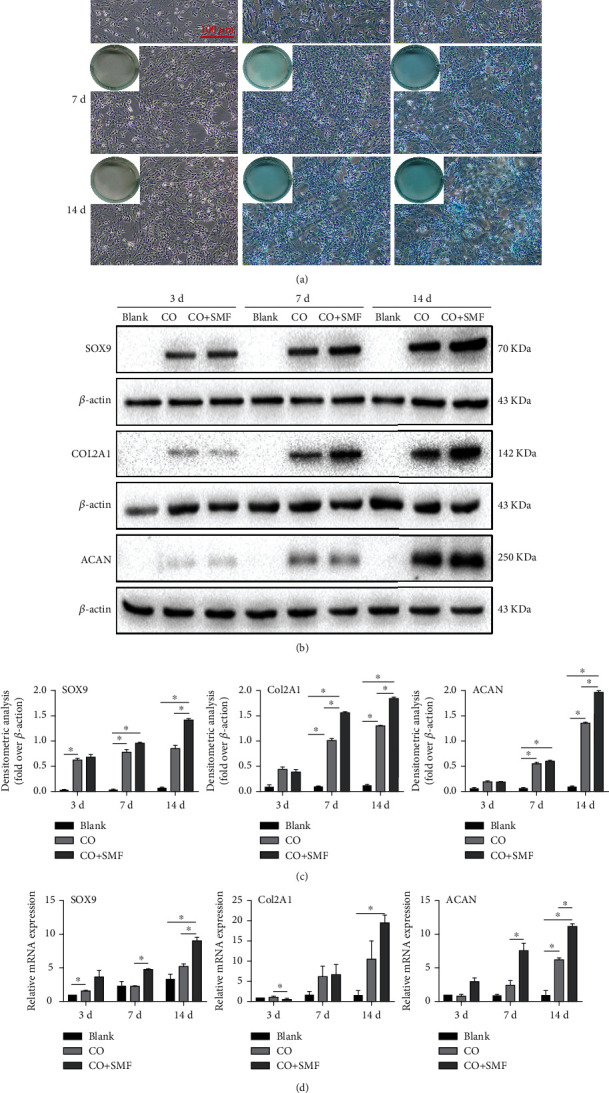
Effect of an SMF on the chondrogenic differentiation of MBMSCs in the coculture system. (a) Comparison of Alcian blue staining of each group. Amplification = 10x, scale bar = 100 *μ*m. (b, c) Western blot analysis of cartilage-related proteins. (d) RT-qPCR analysis of cartilage-related genes. ^∗^*P* < 0.05. Each group repeated three times (*n* = 3). Data presented as means ± SD.

Alcian blue staining showed that the MBMSCs in the blank group were not stained, whereas on day 7, MBMSCs in the experimental group showed a certain degree of aggregation and differentiation, with darker staining. On day 14, the aggregation and differentiation of MBMSCs was more obvious, which exhibited strong positive staining. What is more, the formation of cartilaginous nodules could be observed (Figure 4(a)). Compared with the blank group, western blot analysis showed that the MBMSCs in the control group showed increased SOX9, Col2A1, and ACAN protein expression on days 3, 7, and 14. SOX9 was significantly increased on days 3 and 7 (*P* < 0.05), and Col2A1 and ACAN were significantly increased on days 7 and 14 (*P* < 0.05). SOX9 protein in MBMSCs of the experimental group increased on days 3, 7, and 14, and Col2A1 and ACAN protein expression increased on days 7 and 14. On day 14, the expression of SOX9 and ACAN proteins was significantly elevated (*P* < 0.05). On days 7 and 14, the expression of Col2A1 protein was significantly elevated (*P* < 0.05) (Figures 4(b) and 4(c)). RT-qPCR showed that the expression of *SOX9* and *Col2A1* genes in the control group was increased on days 3 (*P* < 0.05), 7, and 14, and the expression of the *ACAN* gene was increased on days 7 and 14 (*P* < 0.05) when compared to that in the blank group. Compared with the control group, the expression of *SOX9* and *ACAN* genes in the experimental group was increased on days 3, 7 (*P* < 0.05), and 14 (*P* < 0.05), and the expression of *Col2A1* was increased on days 7 and 14 (*P* < 0.05) (Figure 4(d)). These data demonstrate that moderate-intensity SMF increases the content of GAG and the expression of SOX9, Col2A1, and ACAN. It enhances the chondrogenesis of MBMSCs in the coculture system.

## 4. Discussion

The effects of magnetic fields influence biological activities [31], not only in osteoporosis [32] and orthodontics [33] but also with an active role in cartilage tissue engineering [34]. Related studies have provided evidence that moderate-intensity SMF can effectively promote chondrogenic differentiation of BMSCs [10, 25]. Our earlier study tested different SMF strengths by exposing MCCs to 160, 280, or 360 mT and found that the proliferation and chondrogenesis-promoting effects of 280 mT SMF were the most pronounced [34]. In recent years, many studies have attempted to combine SMF with other elements [35]. These studies have not only achieved good results but also provided more insight into how SMF can be used in experimental and clinical settings [36].

The chondrocyte/mesenchymal stem cell coculture approach is reliable and effective for cartilage tissue engineering [37]. Chondrocyte-secreted factors such as bone morphogenetic protein (BMP), TGF-*β*, and insulin-like growth factor-1 (IGF-1) could accelerate chondrogenesis of BMSCs in vitro [38]. TGF-*β*1 activates the Smad signaling pathway through its receptors and initiates cartilage-specific gene transfer, thereby upregulating the expression and synthesis of Col2A1, GAG, and ACAN [39]. Furthermore, cell-derived extracellular vesicles (EVs) include RNA, proteins, DNA, and enzymes [40], and these vesicular contents convey molecular information to recipient cells by receptor-mediated endocytosis or vesicular fusion [41, 42]. Subsequently, these received molecular factors reprogram the activity of recipient cells and affect intercellular communication [43]. Extracellular vesicles mediate intercellular communication in the coculture system, whereby chondrocytes provide a suitable microenvironment for BMSCs, which internalized exosomal miR-8485 released from the chondrocytes and induced chondrogenic differentiation of BMSCs by regulating the Wnt/*β*-catenin signaling pathway [18]. However, the application of this technique is still limited by multiple factors such as stem cell sources, proliferation, and differentiation [44]. There is an urgent need to improve cell coculture conditions to increase the number of seed cells and ameliorate the cartilage phenotype. Therefore, the present study exploited the advantages of moderate-intensity SMF to improve the proliferation and chondrogenesis of MBMSCs in the coculture system.

The results of the proliferation assay showed that moderate-intensity SMF promoted the proliferation of MBMSCs in the coculture system. We conclude that moderate-intensity SMF combined with coculture expands the number of MBMSCs for cartilage tissue engineering. We also found that growth inhibition of MBMSCs under coculture conditions occurred on day 11, which is consistent with previous studies. Zhao et al. [45] obtained similar results (a decrease in the number of BMSCs and a lower survival rate) when cocultured BMSCs and chondrocytes were implanted in a rat knee injury model and tracked by fluorescent protein labeling. We speculate that this may be due to some apoptotic factors secreted by chondrocytes [46], but further confirmation is needed.

During the differentiation of BMSCs into chondrocytes, on day 7, BMSCs initially acquired chondrocyte morphological and functional characteristics and started to aggregate to form monolayers. On day 14, larger multilayer aggregates were formed, and the formation of chondrocyte nodules could be observed [47]. In this process, Col2A1, GAG, and ACAN are key components of the cartilage matrix [48], and SOX9 is a key transcription factor for cartilage development, maintenance of the cartilage phenotype, and chondrogenic differentiation of BMSCs [49]. In agreement with previous studies, our results showed that moderate-intensity SMF enhanced the expression of chondrogenic regulators (SOX9) and cartilage matrix proteins (GAG, ACAN, and Cola2) in MBMSCs under coculture conditions and promoted the chondrogenic differentiation of MBMSCs. Thus, it seems that the moderate-intensity SMF combined with a coculture approach can improve the cartilage phenotype of MBMSCs for cartilage tissue engineering.

In summary, moderate-intensity SMF significantly enhanced the chondrogenesis and proliferation of MBMSCs in the coculture system, and it might be a promising approach to repairing condyle cartilage defects in the clinical setting. This technique could be used to rapidly expand the MBMSCs which have improved the cartilage phenotype before constructing 3D tissue engineering cartilages. Then, it is implanted into scaffolds with good biocompatibility that could be gradually degraded and absorbed in the body. Finally, the composite cartilage material is transplanted to the damaged cartilage site to achieve the purpose of repairing the condylar cartilage defect.

## Data Availability

The data used to support the findings of this study are available from the corresponding author upon request.
